# Adolescent pilonidal disease laser treatment (a-PiLaT): a pilot study

**DOI:** 10.1007/s10151-024-02972-w

**Published:** 2024-08-14

**Authors:** A. Romanova, M. Nissen, M. Alrefai, J. Hubertus, T. Deska, M. Senkal

**Affiliations:** 1 Department of Pediatric Surgery, Marien Hospital, St. Elisabeth Gruppe, Ruhr-University of Bochum, Marienplatz 2, 58452 Witten, Germany; 2 Department of Surgery, Marien Hospital, St. Elisabeth Gruppe, Marienplatz 2, 58452 Witten, Germany

**Keywords:** Pilonidal disease, Pilonidal cyst, Laser, Adolescent surgery

## Abstract

**Background:**

Pilonidal disease (PD) is an acquired condition related to hair-induced mechanical forces on the skin surface of the intergluteal cleft, with subsequent abscess formation with or without a concomitant draining sinus (pit). While surgical management currently is the mainstay of treatment, pilonidal disease laser treatment (PiLaT) has recently been recognized as a promising treatment option for non-inflammatory diseases. Nonetheless, there is a paucity of available data on adolescent pilonidal disease laser treatment (a-PiLaT).

**Methods:**

We describe our preliminary experience with PiLaT performed in adolescents aged 10–17 years at our tertiary paediatric surgical hospital from 2019 to 2023. Data on perioperative characteristics and clinical outcomes at follow-up were retrospectively analysed.

**Results:**

A total of 17 consecutive patients (*n* = 12 female, 71%) underwent a-PiLaT. At the time of treatment, the patients’ mean age and body mass index were 13.6 ± 1.6 years and 25.3 ± 5.6 kg m^−2^, respectively. The mean operative time was 21.5 ± 10.4 min, whereas the mean follow-up period was 24.5 ± 16.8 months, with a complication rate of 24% (*n* = 4) and recurrence rate of 18% (*n* = 3). With respect to postsurgical scar assessment, the mean Patient and Observer Scar Assessment Scale scores (score range 6–60, with higher scores indicating worse outcome) were 14.2 ± 6.5 (patients’ evaluation) and 11.4 ± 4.7 (observers’ evaluation).

**Conclusion:**

The a-PiLaT represents a novel approach for managing PD in adolescents. Our preliminary data on the outcomes of a small series of patients with pilonidal sinuses after a-PiLaT indicated complication and recurrence rates comparable to those reported in the literature for adults. This new minimally invasive technique has great potential and is therefore worthy of further research on a larger population.

## Introduction

Pilonidal disease (PD) was first described almost 200 years ago by Mayo [1]. Regarding its aetiology, a foreign body reaction secondary to broken or overturned hair in the buttocks or natal cleft may be an initiating factor for PD [2]. Another theory suggests that pits develop as a result of the clogging and distension of keratin hair follicles [3, 4]. PD typically occurs in young people aged 15–30 years, with an incidence of 26/100,000. Previous studies have described the role of hormonal factors in relation to the onset of this disease during the postpubertal period [5] and have identified obesity, male sex, sedentary lifestyle and hairiness as risk factors [6–8]. Another study also showed that the occurrence of PD in teenagers was influenced by heredity and the frequency of weekly baths [9]. Recently, some risk factors associated with disease development have been reviewed. For instance, a study using pilocarpine iontophoresis to assess sweating in the glabella sacralis revealed that patients with a pilonidal sinus showed less sweating, suggesting that sweating might be a protective factor rather than a risk factor for PD [10].

To date, many treatment options are available for this disease. Nevertheless, there exists no recognised gold standard, and the optimal mode of treatment for PD remains unknown. The most common surgical techniques include excision with or without primary closure, excision and packing, different flap procedures, marsupialisation, laser therapy and thrombin-gelatin matrix injection. In the last decade, new treatment options such as endoscopic pilonidal sinus treatment [11] and crystallised phenol application [12] have been explored. In children, laser therapy in the form of laser hair removal is often employed during the preparatory stage before or after surgery to prevent recurrence [11, 13]. Herein, we described the use of laser therapy for pilonidal sinus ablation as a primary treatment option for PD in adolescents. Considering the active lifestyle of this cohort, treatment should not lead to prolonged hospitalisation, and the return to normal life should be rapid. We believe that laser therapy has the potential to satisfy these requirements.

## Materials and methods

This retrospective series included 17 patients aged 10–17 years whose pilonidal sinus (ICD-10 L05.9) was treated using adolescent pilonidal disease laser treatment (a-PiLaT) at the Department of Paediatric Surgery of our tertiary paediatric surgical hospital in Germany from 2019 to 2023. In the case of an uncomplicated postoperative period, patients were examined on an outpatient basis at 1 week, 3 weeks and 3–4 months after surgery. The timing of examination for the study patients was not standardised.

Patients who previously underwent simple abscess drainage were included in this study. The exclusion criteria for this study were as follows: PD with signs of inflammation, especially in the presence of secondary infection and abscess formation, and age ≥ 18 years.

The following data were extracted from patient records: sex (male/female), age (years), weight (kg), length (m), body mass index (kg m^−2^; BMI), symptom duration (months), number of pilonidal pits (*n*), a-PiLaT duration (min), complications (yes/no), recurrence (yes/no), follow-up period (months) and Patient and Observer Scar Assessment Scale (POSAS) score [14].

The main outcome measure was the detection of PD recurrence. The Clavien–Dindo classification [15], which consists of seven grades (I, II, IIIa, IIIb, IVa, IVb and V), was used to determine the therapy necessary to correct a specific complication. Two consultant-level surgeons performed all a-PiLaT sessions, one of whom was an expert in the radial laser probe technique and trained the second surgeon.

This study was approved by the Ethics Committee of Ruhr University Bochum (registry no. 21-7397-BR). Informed consent was obtained from all study participants and their parents.

### Surgical technique

**Fig. 1:**
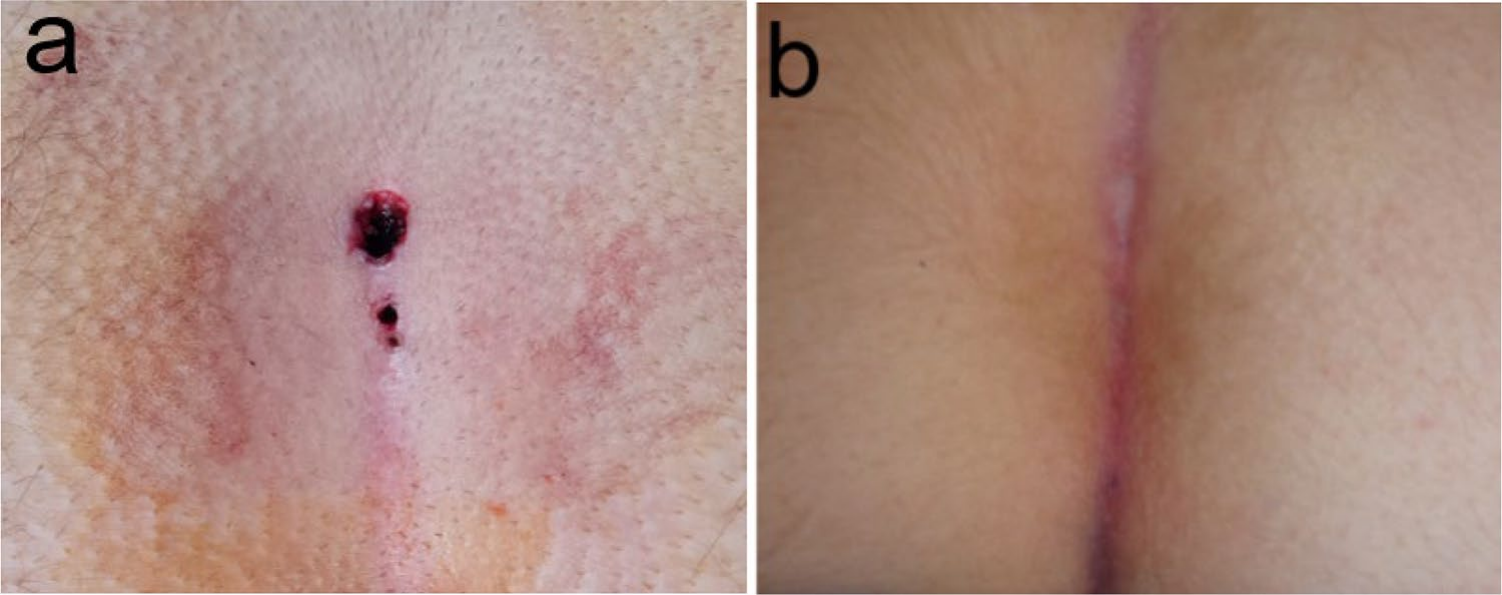
**a** Pilonidal sinus after pit-picking and laser application. **b** Postoperative result after 4 weeks

All procedures were performed with patients in the prone position under general anaesthesia. Before cleaning was performed with povidone-iodine, hair was removed from the surgical field using a razor. The buttocks were moved apart using adhesive tape attached to the sides of the torso to provide easy access to the surgical field. The external openings of sinuses were then excised using a scalpel with a 1-mm skin margin (“pit-picking”), and the deep content of the sinus was removed using a mosquito clamp. Subsequently, the sinus was washed with a saline solution. The subcutaneous tissue around the pits was infiltrated with the saline solution to avoid burning the skin. The laser (Diodenlaser LEONARDO® DUAL 45; Biolitec, Jena, Germany) was introduced into the sinus up to its end, followed by its activation. Afterward, it was slowly retracted at a speed of 5 mm/s. The power applied during each procedure was 7 W, and the procedure was repeated for each pit. The external openings were not closed (Fig. 1a). After discharge, epithelialisation was monitored during follow-up visits (Fig. 1b).

### Statistical analysis

Sampling and statistical analysis of data were performed using Microsoft Excel version 2308 (Microsoft Corporation, Redmond, WA, USA). The results are expressed as means ± standard deviations (SDs) and, where indicated, as range (min–max).

## Results

Tables 1 and 2 summarise the biometric and procedural characteristics. A total of 17 consecutive patients (*n* = 12 females 71%) underwent laser therapy for PD. At the time of treatment, the patients’ mean age and BMI were 13.6 ± 1.6 years and 25.3 ± 5.6 kg m^−2^, respectively. The mean duration of symptoms was 1.8 ± 1.6 months, whereas the mean follow-up period was 24.5 ± 16.8 months.

**Table 1:** Overview of patient characteristics

Patient	Age [years]	Sex [F/M]	BMI [kg m^−2^]	Symptoms duration [months]	Pits [*n*]	Procedure time [mins]	Previous surgery [Y/N]	Complications [Y/N]	Recurrence [Y/N]	CDC [grade]	Measurement time after surgery [months]	Follow-up [months]	POSAS (patient)	POSAS (observer)
1	13	F	20	2	1	13	Y	N	N	–	0.5	9	15	16
2	10	F	21.5	1	2	13	N	Y	N	I	18	46	8	6
3	12	F	39.3	1	1	19	N	N	N	–	10	38	14	9
4	14	F	22.7	1.5	2	42	N	Y	N	IIIa	17	44	16	10
5	13	F	23	7	2	29	N	Y	Y	II	3	30	23	18
6	13	M	30.1	3	2	31	Y	Y	Y	I	6	26	24	8
7	12	F	26	0.5	5	11	N	N	N	–	6	45	26	10
8	14	F	27.9	4	2	38	Y	N	N	–	3	3	7	7
9	13	F	20	2	2	15	Y	N	N	–	13	38	23	22
10	14	M	20	1	3	15	Y	N	Y	–	23	50	13	20
11	14	M	18.6	0.5	2	2	N	N	N	–	6	33	11	11
12	17	M	21.4	1	2	30	N	N	N	–	13	13	10	10
13	14	F	29	0.5	3	27	Y	N	N	–	11	11	8	10
14	16	F	34.8	0.75	1	17	Y	N	N	–	5	5	12	10
15	15	F	23.9	1	2	17	Y	N	N	–	9	9	7	10
16	14	M	26.4	2	1	25	N	N	N	–	7	7	7	9
17	14	F	25	2	2	21	N	N	N	–	10	10	17	8

*F* female, *M* male, *BMI* body mass index, *Y* yes, *N* no, *CDC* Clavien–Dindo classification, *POSAS* Patient and Observer Scar Assessment Scale scores

**Table 2:** Literature data on biometric, technical and procedural parameters for the use of laser treatment for the pilonidal sinus

Study	Type of study	Study period	Cohort size (*n*)	Mean age [years] (min–max)	Male/female ratio	Laser type (wavelength; power)	Anaesthesia type	Mean surgery duration [min] (min–max)	Mean follow-up period [m] (min–max)	Overall complication rate (%)	Recurrence rate (%)
Dessily et al. [17]	Monocentric retrospective	09/2014–09/2015	40	25.2 (15–46)	33/7	1470 nm; 10 W	Spinal, general	NA	7 (3–10)	10	2.9
Dessily et al. [18]	Monocentric prospective	03/2015–08/2017	200	24.5 ± 7.2 (15–45)	72/28	1470 nm; 10 W	Usually spinal	9.4 ± 2.6 (6–16)	17 (6–37)	15	14.9
Harju et al. [19]	Monocentric retrospective	01/2017–09/2019	86	29.2 (18–67)	68/18	1470 nm; 10 or 13 W	Local 85%; general 5%; spinal 10%	NA	14 (6–37)	13.9	3
Porwal et al. [20]	Monocentric prospective	01/2013–12/2017	228	27.16 ± 8.06 (13–68)	200/28	1470 nm diode laser; 8 W	Local	33.32 ± 6.49 (12–65)	12	15.4	2.6
Yardimci et al. [21]	Monocentric comparative randomized	01/2016–01/2018	30	27.9 ± 8.9 (15–49)	25/5	1470 nm diode laser; 12–14 W	General	15.1 ± 2.0 (12–20)	25 (15–39)	3.3	3.3
Georgiou [22]	Monocentric prospective	04/2015–12/2016	60	22.7 ± 6.23 (15–58)	51/9	1470 nm diode laser; 8 W	Local	32.3 ± 8.12 (23–65)	12	23.2	8,3
Our study	Monocentric retrospective	12/2019–09/2023	17	13.6 ± 1.6 (10–17)	5/12	1470 nm, 7 W	General	21.5 ± 10.4 (2–42)	24.5 ± 6.8 (3–50)	24	18

*NA* not available, *W* watts, *m* months

Prior to the PiLaT procedure, eight (47%) patients underwent surgery for pilonidal sinus abscess formation. The mean operative time was 21.5 ± 10.4 min, whereas the mean number of pits was 2 ± 1.

Postprocedural complications occurred in four (24%) patients, including local infections (*n* = 2, 12%) and wound healing disorders with or without prolonged wound secretion (*n* = 2; 12%). Postoperative complications were graded according to the Clavien–Dindo classification (Table 1). Prolonged wound secretion in two (12%) patients who received conservative therapy with silver nitrate was classified as grade I. One (6%) patient with local infection (grade II) was treated with oral antibiotics, and the abscess (grade IIIa) in another one (6%) patient was drained under local anaesthesia. Of note, we did not classify serous wound discharge as a complication during the first 6 weeks postoperatively.

Overall, recurrence was detected in three (18%) patients. Among these patients, two (12%) previously experienced postoperative complications. Furthermore, two (12%) patients underwent revision of the a-PiLaT procedure, with good results, whereas the other one (6%) patient underwent the Karydakis procedure in another hospital. Reoperations were performed at 12.7 ± 3.8 months later. Compared with the BMI of patients without any complications (25.5 ± 5.9; *n* = 14), the BMI of patients with postprocedural complications (24.3 ± 3.9; *n* = 4) and patients requiring redo procedure (24.4 ± 5.2, *n* = 3) did not differ from each other (*P* = 0.93 and *P* = 0.95).

With respect to postsurgical scar assessment, the mean POSAS scores (score range 6–60, with higher scores indicating worse outcome) were 14.2 ± 6.5 (patients’ evaluation) and 11.4 ± 4.7 (observers’ evaluation). Four (24%) patients (namely, patients 5, 6, 7 and 9) had a POSAS score of ≥ 20. Patient 7 complained of relevant itching. Patients 5 and 6 disliked the appearance of the scar, particularly its colour, thickness and/or irregularity. Patient 9 was particularly dissatisfied with the colour of the scar. Patients 1, 5, 7 and 14 had moderate pain. Among patients with a POSAS score of ≥ 20, patients 5 and 6 experienced PD recurrence, patient 9 previously underwent surgery for PD, and patient 7 had an uneventful course. The observers rated the POSAS score as ≥ 18 for patients 5, 9 and 10. The predominant parameters leading to poor ratings were relief and surface area.

In this study, we also calculated the sacral-lumbar skin distension (SL) quotient, which was first described by Dahmann et al. [16] and is independent of age and BMI (standard range approximately 0.8–0.93) [16]. In our study, the mean SL quotient was 0.84 ± 0.14, with patient 3 having the lowest SL quotient (0.67). Seven patients (namely, patients 4, 9, 11, 12, 13, 15 and 17) had an SL quotient within the normal range for healthy skin. None of the patients with a history of abscess drainage prior to laser treatment had an SL quotient of < 0.7.

## Discussion

The first application of lasers in the field of medical technology occurred immediately after their invention for medical indications in the 1960s. Laser therapy is currently actively applied in paediatric surgery to treat infantile haemangiomas, vascular malformations, bezoars, kidney and biliary stones, scars and pyogenic granulomas [13]. Moreover, local laser application has been successfully implemented in adult pilonidal sinus treatment (Table 2) [17–22].

In our retrospective series, we described our first experience with a-PiLaT. Unfortunately, there is a dearth of published studies in the literature regarding the application of this procedure in adolescents. For instance, Fernandes et al. compared two methods for treating PD, one of which involved pit-picking with laser ablation [23]. In their investigation, 36 patients underwent pit-picking with laser ablation. Notably, the patients’ age ranged from 15 to 16 years, whereas our study included patients aged 10–17 years. While their surgical technique mirrored ours, the authors only provided the laser wavelength, omitting the laser power details. Our complication rate was 7% higher, yet the recurrence rate remained comparable. Moreover, we juxtaposed our findings with published outcomes derived from adult populations (Table 2). In the present study, most patients were female (71%), which differs from the findings of previous studies (Table 2). Although it is generally accepted that men are more prone to PD, the number of women diagnosed with PD has recently increased [24]. Interestingly, the ratio between affected male and female individuals has remained constant. The reason for the increase in the number of women with PD in our adolescent cohort is unclear. Given that obesity is a risk factor for PD development, our observed female preponderance cannot be explained by an increase in the frequency of adolescents with obesity because this cohort is also predominantly male [25]. This may have been biased owing to our small cohort.

The technique applied was almost the same as that described in similar articles. In contrast to the centres listed in Table 2, we performed this procedure under general anaesthesia. While the use of another type of anaesthesia is uncommon in paediatric surgery owing to the young age of patients, there are cases in which the operation was performed under local anaesthesia with mild sedation [23]. In our study, the mean operative time was 21.5 ± 10.4 min (range 2–42 min), and the complication rate was 24% (*n* = 4), which was higher than that in other medical centres (3.3% [21] and 15.4% [20]) (Table 2). The most common complications in the present study were prolonged wound secretion and infection. Our patients did not have haematomas or seromas, which have been reported after similar procedures in adults [17, 22]. The recurrence rate was also high at 18% (*n* = 3). One possible explanation might be the use of a lower-power (7-W) diode laser at a wavelength of 1470 nm, as compared with the higher power levels (range 8–14 W) applied in the adult cohort (Table 2).

The POSAS score was used to categorise the postoperative cosmetic outcomes after a-PiLaT. This score helps in evaluating different scar characteristics (pain, itching, colour, stiffness, thickness and irregularity) and assessing patients’ overall satisfaction with the results. In general, the patients were satisfied with the cosmetic outcomes of the procedure. Additionally, we used a novel method for measuring sacral skin elasticity, as described by Dahmann et al. [26]. They used the SL quotient to compare secondary healing (SL quotient of 0.75) to Limberg flap wound closure (SL quotient of 0.86) after pilonidal sinus excision. In the healthy group, the SL quotient was 0.87. In our study, the mean SL quotient was 0.84. Our results indicated that after a-PiLaT, scar tissues formed, and the skin became less elastic. Because laser treatment is minimally invasive, we were surprised by the results, which are similar to those obtained after flap surgery.

Laser treatment is not the only technique that should be considered for treating PD in children and adolescents. A systematic review of surgical interventions in the paediatric population showed that the best outcomes for recurrence rates (6–7%) were achieved with midline primary closure, marsupialisation and minimally invasive techniques [27]. Minimally invasive methods such as pit-picking [28], pit-picking with laser ablation [23] and paediatric endoscopic pilonidal sinus treatment (PEPSiT) [29] are gaining popularity in paediatric and adolescent surgery. In our study, the recurrence rate was 18%, which is similar to the results reported by Fernandes et al. in 36 patients [23], in which the recurrence rate was 17% after a similar procedure. In comparison, the recurrence rate after PEPSiT was only 4.6% [30]. Another notable advantage of PEPSiT is the wealth of experience documented in performing the procedure under saddle spinal block or locoregional anaesthesia [30].

We showed that the a-PiLaT procedure is feasible in adolescents without major complications. However, despite the overall satisfactory cosmetic results reported by the patients, we observed a relatively high number of recurrences and complications. This study had two limitations: one is the increasing learning curve of the technique itself, and the other is the relatively small number of patients treated. Because cosmetic results and patient satisfaction favour the a-PiLaT procedure, we still need to improve the technical aspects and the procedure itself. However, with the ongoing acquisition of patients, special attention should be paid to the outcomes. Should it become apparent that even adjustments to the technique will not result in significant improvements, further use of a-PiLaT will have to be critically discussed.

## Conclusions

Laser therapy may constitute a novel approach for managing adolescent patients with PD. Currently, we are inspired by the results and plan to continue using this method, given that the cosmetic results and patient satisfaction support the use of a-PiLaT. We plan to perform laser ablation under local anaesthesia in the future based on the experience of our colleagues who have performed similar operations. We are also considering changes in the technical aspects of this operation, such as the power of the laser probe, as well as the use of hair removal in the preparatory and postoperative phases. Should it become apparent that even adjustments to the technique will not result in significant improvements, further use of a-PiLaT will have to be critically discussed. We consider PEPSiT as an alternative method for treating PD, which has proven itself very well.

## Data Availability

The raw data supporting the conclusions of this article will be made available by the authors without undue reservation.

## References

[1] Mayo OH (1833) Observations on injuries and diseases of the rectum. Burgess and Hill, London PMC5086971 29918031

[2] Karydakis GE (1992) Easy and successful treatment of pilonidal sinus after explanation of its causative process. Aust N Z J Surg 62:385–389 1575660 10.1111/j.1445-2197.1992.tb07208.x

[3] Bascom J (1983) Pilonidal disease: long-term results of follicle removal. Dis Colon Rectum 26:800–807 6641463 10.1007/BF02554755

[4] Bascom J (1994) Pilonidal sinus. Curr Pract Surg 6:175–180

[5] Özkan Z, Aksoy N, Emir S (2014) Investigation of the relationship between serum hormones and pilonidal sinus disease: a cross-sectional study. Colorectal Dis 16(4):311–314 24330514 10.1111/codi.12520

[6] Stelzner F (1984) Die Ursache des Pilonidalsinus und der Pyodermia fistulans sinifica. Langenbecks Arch Chir 362:105–118 6738258 10.1007/BF01254185

[7] Jones DJ (1992) Pilonidal sinus. BMJ 305:410–412 1392926 10.1136/bmj.305.6850.410 PMC1883113

[8] Franckowiak JJ, Jackman RJ (1962) The etiology of pilonidal sinus. Dis Colon Rectum 5:28–36 13894530 10.1007/BF02616408

[9] Yildiz T, Elmas B, Yucak A, Turgut HT, Ilce Z (2017) Risk factors for pilonidal sinus disease in teenagers. Indian J Pediatr 84(2):134–138 27306225 10.1007/s12098-016-2180-5

[10] Doll D, Brengelmann I, Schober P (2021) Rethinking the causes of pilonidal sinus disease: a matched cohort study. Sci Rep 11(1):6210 33737662 10.1038/s41598-021-85830-1 PMC7973489

[11] Esposito C, Mendoza-Sagaon M, Del Conte F (2020) Endoscopic pilonidal sinus treatment (PEPSiT) in children with pilonidal sinus disease: tips and tricks and new structurated protocol. Front Pediatr 8:345 32671004 10.3389/fped.2020.00345 PMC7326782

[12] Arslan S, Okur MH, Basuguy E (2021) Crystallized phenol for treatment of pilonidal sinus disease in children: a comparative clinical study. Pediatr Surg Int 37(6):807–813 33856512 10.1007/s00383-020-04798-7

[13] Kopeć J, Przewratil P (2020) Laser therapy in pediatric sugery. Pediatria I Medycyna Rodzinna 16(2):165–170

[14] van de Kar AL, Corion LU, Smeulders MJ, Draaijers LJ, van der Horst CM, van Zuijlen PP (2005) Reliable and feasible evaluation of linear scars by the patient and observer scar assessment scale. Plast Reconstr Surg 116(2):514–522 16079683 10.1097/01.prs.0000172982.43599.d6

[15] Dindo D, Demartines N, Clavien PA (2004) Classification of surgical complications: a new proposal with evaluation in a cohort of 6336 patients and results of a survey. Ann Surg 240:205–213 15273542 10.1097/01.sla.0000133083.54934.ae PMC1360123

[16] Dahmann S, Lebo PB, Görlich D (2016) Sacral skin elasticity–establishing a non-invasive mechanical method for measurement. Handchir Mikrochir Plast Chir 48(04):212–218 27547929 10.1055/s-0042-104118

[17] Dessily M, Charara F, Ralea S, Allé JL (2017) Pilonidal sinus destruction with a radial laser probe: technique and first Belgian experience. Acta Chir Belg 117(3):164–168 28056720 10.1080/00015458.2016.1272285

[18] Dessily M, Dziubeck M, Chahidi E, Simonelli V (2019) The SiLaC procedure for pilonidal sinus disease: long-term outcomes of a single institution prospective study. Tech Coloproctol 23(12):1133–1140 31773347 10.1007/s10151-019-02119-2

[19] Harju J, Söderlund F, Yrjönen A, Santos A, Hermunen K (2021) Pilonidal disease treatment by radial laser surgery (FiLaC™): the first Finnish experience. Scand J Surg 110(4):520–523 33349142 10.1177/1457496920975610

[20] Porwal A, Gandhi P, Kulkarni D (2020) Laser pilonidotomy—a new approach in management of complex pilonidal sinus disease: an exploratory study. JCOL 40(01):024–030

[21] Yardimci VH (2020) Outcomes of two treatments for uncomplicated pilonidal sinus disease: Karydakis flap procedure and sinus tract ablation procedure using a 1470 nm diode laser combined with pit excision. Lasers Surg Med 52(9):848–854 32064640 10.1002/lsm.23224

[22] Georgiou GK (2018) Outpatient laser treatment of primary pilonidal disease: the PiLaT technique. Tech Coloproctol 22(10):773–778 30306277 10.1007/s10151-018-1863-5

[23] Fernandes S, Soares-Aquino C, Teixeira I, Monteiro JM, Campos M (2022) Minimally invasive treatment of pilonidal sinus disease in a paediatric population: comparison of two techniques. ANZ J Surg 92(12):3288–3292 35678224 10.1111/ans.17838

[24] Luedi M, Schober P, Stauffer V (2021) Gender-specific prevalence of pilonidal sinus disease over time: a systematic review and meta-analysis. ANZ J Surg 91(7–8):1582–1587 34101331 10.1111/ans.16990

[25] Mensink GBM, Schienkiewitz A, Haftenberger M (2013) Übergewicht und Adipositas in Deutschland. Bundesgesundheitsbl 56:786–794 10.1007/s00103-012-1656-3 23703499

[26] Dahmann S, Lebo PB, Meyer-Marcotty MV (2016) Comparison of treatments for an infected pilonidal sinus: differences in scar quality and outcome between secondary wound healing and Limberg flap in a prospective study. Handchir Mikrochir Plast Chir 48:111–119 27096210 10.1055/s-0041-111322

[27] Hardy EJO, Herrod PJ, Doleman B, Phillips HG, Ranat R, Lund JN (2019) Surgical interventions for the treatment of sacrococcygeal pilonidal sinus disease in children: a systematic review and meta-analysis. J Pediatr Surg 54(11):2222–2233 30940347 10.1016/j.jpedsurg.2019.02.058

[28] Delshad HR, Dawson M, Melvin P, Zotto S, Mooney DP (2019) Pit-picking resolves pilonidal disease in adolescents. J Pediatr Surg 54(1):174–176 30661599 10.1016/j.jpedsurg.2018.10.021

[29] Esposito C, Izzo S, Turrà F et al (2018) Pediatric endoscopic pilonidal sinus treatment, a revolutionary technique to adopt in children with pilonidal sinus fistulas: our preliminary experience. J Laparoendosc Adv Surg Tech A 28(3):359–363 29232530 10.1089/lap.2017.0246

[30] Esposito C, Montaruli E, Autorino G, Mendoza-Sagaon M, Escolino M (2021) Pediatric endoscopic pilonidal sinus treatment (PEPSiT): what we learned after a 3-year experience in the pediatric population. Updat Surg 73(6):2331–2339 10.1007/s13304-021-01094-4 PMC8606398 34021885

